# Human Papillomavirus in Non-Small Cell Lung Carcinoma: Assessing Virus Presence in Tumor and Normal Tissues and Its Clinical Relevance

**DOI:** 10.3390/microorganisms11010212

**Published:** 2023-01-14

**Authors:** Matvey M. Tsyganov, Marina K. Ibragimova, Evgeniy O. Rodionov, Olga V. Cheremisina, Sergei V. Miller, Sergei A. Tuzikov, Nikolai V. Litvyakov

**Affiliations:** 1 Cancer Research Institute, Tomsk National Research Medical Center, Russian Academy of Sciences, 5 Kooperativny Street, 634050 Tomsk, Russia; 2 Biological Institute, The National Research Tomsk State University, 36 Lenin Ave., 634050 Tomsk, Russia

**Keywords:** human papillomavirus, HPV, non-small cell lung cancer, viral load, physical status, metastatic-free survival

## Abstract

The significance of the role of human papillomavirus (HPV) in the development of lung cancer remains an open question. The data from the literature do not provide conclusive evidence of HPV being involved in the pathogenesis of lung cancer. The aim of this work was to detect the presence of HPV infections with a high carcinogenic risk in patients with non-small cell lung cancer (NSCLC). Materials and methods: the study involved 274 patients with stage IIA–IIIB non-small cell lung cancer. We analyzed normal and tumor tissues as well as blood from each patient. DNA was extracted from patients’ specimens, and HPV detection and genotyping was carried out using commercially available kits by PCR. Results: HPV was detected in 12.7% of the patients (35/274 of all cases). We detected nine different types of human papillomavirus in the patients, namely, types 16, 18, 31, 35, 45, 51, 52, 56, and 59. The HPV-positive samples had a clinically insignificant viral load and were predominantly integrated. The relationship between the presence of HPV and its virological parameters and the clinical and pathological parameters of the patients was established. A metastatic-free survival analysis showed that all patients with HPV in the tumor tissue had a higher 5-year survival rate (94%) compared with the HPV-negative patients (78%). The result was not statistically significant (*p* = 0.08). Conclusions: data showing a 12.7% human papillomavirus representation among patients with non-small cell lung cancer were obtained. The presence/absence of a viral component in patients with lung cancer was a clinically significant parameter. HPV types 16, 18, and 56, which are the most oncogenic, were most often detected.

## 1. Introduction

Many factors play a role in lung cancer (LC), ranging from smoking and unfavorable environmental conditions to various individual molecular genetic parameters of the organism. The possible cause of the development of LC may be polymorphisms in the genes that regulate the metabolism of carcinogenic substances, the cell cycle, and other key processes in carcinogenesis, for example [1,2]. There is also the hypermethylation of the promoters of various genes associated with important functions in lung cancer, including the control of proliferation, apoptosis, cell adhesion, and DNA repair [3,4]. However, the viral etiology of the development of lung cancer has not been considered. The influence of viruses on the course of this malignant neoplasm has not been confirmed or refuted. Only a few papers have focused on human papillomavirus (HPV). HPV is an important etiological and prognostic factor not only for gynecological pathologies of varying severity [5] but also for tumors and other tumor localizations, in particular, the upper respiratory tract and the digestive tract [6].

A few authors have described cases of a 100% spontaneous transformation of papillomas into differentiated squamous cell carcinomas of the lungs (51.1%) and the larynx (42.9%) [7].

HPVs with a low carcinogenic risk (LCR) (types 6 and 11) were previously identified in papillomas in such patients. According to meta-analysis data, the frequency of occurrence of human papillomavirus in LC widely varies, depending on the number of patients examined, from a complete absence of the virus in the tumor tissue to an occurrence in more than 75% of cases [8,9]. In most cases, HPV has a high carcinogenic risk, most notably types 16 and 18, which are currently recognized in the global literature as leaders in the frequency of occurrence of cervical cancer and other solid tumors [10].

In a recent study, Rezaei et al. showed an association between HPV and lung cancer [11]. In particular, the authors found the presence of HPV DNA in 52.9% (54/102) of the patient samples and in 25% (12/48) of the control samples. A significant association was observed between an HPV-positive status and lung cancer (OR = 3.37; 95% CI = 1.58–7.22; *p* = 0.001). The most prevalent virus genotype in the patients was type 16 (38.8%). A decrease in the p53 and RB expression was shown; the expression of inflammatory cytokines was increased in the HPV-positive lung cancer samples and control tissues compared with the HPV-negative lung cancer and HPV-negative control tissues. As a result, the authors suggested that an HPV infection could cause the induction of inflammation and EMT, which may contribute to the development of lung cancer.

Despite the availability of such work, the question of the role of HPV in the pathogenesis of lung cancer remains open and requires discussion. The aim of this work was to detect the presence of HPV infections with a high carcinogenic risk in patients with non-small cell lung cancer (NSCLC).

## 2. Materials and Methods

The study involved 274 patients with primary operable non-small cell lung cancer (IIB-IIIA stage), central and peripheral localization, aged 39–76, with an average age of 60.8 years old, undergoing treatment at the Cancer Research Institute, Tomsk NRMC, from 2010 to 2016. The study was conducted according to the ethical principles suggested in the Declaration of Helsinki (fixed in 2013) and approved by the Ethical Committee of the Cancer Research Institute (Protocol No. 1, dated 15 January 2016). The diagnosis was verified histologically. The patients were conditionally divided into two study groups, depending on the treatment tactics: a group of patients with neoadjuvant chemotherapy (NAC) (*n* = 126) and a group of patients with primary resectable non-small cell lung cancer (*n* = 148). Patients in the first group received two courses of neoadjuvant chemotherapy according to the vinorelbine 25 mg/m^2^ (1st and 8th days)/carboplatin AUC 6 (2nd day) regimen. The administration of neoadjuvant chemotherapy is determined by the large size of the primary tumor node and the presence of lymph node metastases. The main objective of neoadjuvant chemotherapy is to damage tumor cells, reduce tumor size, reduce the degree of malignancy, and sanitize the lymphatic channels of the lung root and mediastinum to prevent lymphatic and hematogenous metastases and, therefore, relapse and the possibility of organ-preserving surgery. After NAC, these patients underwent surgery in the form of a pneumonectomy or lobectomy. The second group of patients did not receive NAC and were immediately operated on for pneumonectomy and lobectomy.

After the operation, all patients received three courses of adjuvant chemotherapy (ACT) with «platinum doublets» according to the following schemes: vinorelbine 25 mg/m^2^ (1st and 8th days)/carboplatin AUC 6 (2nd day); doxorubicin 50 mg/m2/carboplatin AUC 6 (on day 2); gemcitabine 1250 mg/m^2^ (1st and 8th days)/carboplatin AUC 6 (2nd day); carboplatin AUC6 (on day 2)/paclitaxel 175 mg/m^2^. The interval between chemotherapy courses was 3 weeks. Chemotherapy was administered to patients who were in good general condition and had normal laboratory parameters. The research was conducted in accordance with the 1964 Helsinki Declaration and the local ethics committee of the institute; all patients signed informed consents for the study. 

In addition, the control group, which consisted of 30 apparently healthy volunteers (11 women and 19 men), was included in the study. Average age: 58.1 ± 1.05 (within 47–69 years). The main clinical and pathological parameters of patients are presented in Table 1.

### 2.1. Material

The test materials used as biopsy samples of normal and tumor tissue before treatment (~10–15 mm^3^), taken by bronchoscopy, were surgical samples of normal lung tissue, normal bronchial tissue, tumor tissue (~60–70 mm^3^), and whole blood. Tissue samples were placed in an RNAlater solution (Thermo Scientific, Waltham, MA, USA) and stored at −80 °C (after a 24-h incubation at +4 °C) for further DNA isolation. Blood was collected in vacuum tubes with K2 EDTA (Becton Dickinson, Franklin Lakes, NJ, USA). The sampling of bronchoalveolar lavage to form a control group was performed using fibrobronchoscopy. Whole blood samples were also taken from this group.

### 2.2. DNA Extraction

DNA was isolated using the QIAamp DNA mini Kit (Qiagen, Hilden, Germany). DNA concentration and purity of isolation were evaluated on Qubit 4.0 (Thermo Scientific, Waltham, MA, USA) using a DNA quantification kit. The concentration ranged from 10 to 100 ng/μL. The isolated DNA was stored at −80 °C in a low-temperature freezer (Sanyo, Osaka, Japan).

### 2.3. Definition and Typing of HPV

The detection and genotyping of HPV DNA were performed by real-time PCR on a RotorGene 6000 (Qiagen, Hilden, Germany) using Amplisens^®^ reagent kits (Amplisens^®^ HPV VKR screen-titer-FL, cat #R-V31- T4x (RG, iQ, Mx); AmpliSens^®^ HPV HR genotype FL, cat #R-V25 (RG, iQ, Mx) (Amplisens, Moscow, Russia). The presence of HPV high-risk (HR) 16, 18, 31, 33, 35, 39, 45, 51, 52, 56, 58, and 59 genotypes was determined. The value of viral load was calculated in genomic equivalents of HPV DNA/10^5^ cells, and the threshold of the relevant amount of the virus was taken to be equal to 3 lg HPV DNA/10^5^ cells. The method is based on simultaneous amplification (multiplex-PCR) and “real-time” detection of DNA regions E1-E2 of HPV genes and a beta-globin gene used as an endogenous internal control. To exclude false-positive and false-negative results, each sample was duplicated during PCR.

### 2.4. Digital PCR

For additional verification of the HPV infection in HPV-positive samples (only 16 and 18 types), digital PCR was used (Droplet Digital PCR-System ddPCR QX200, (Bio-Rad, Hercules, CA, USA). DNA (100 ng) was cleaved using HaeIII (SibEnzyme, Novosibirsk, Russia). The mastermix for ddPCR included 1× ddPCR Supermix for probes (no dUTP, BIO-RAD), 0.9 μM primer, and 0.25 μM probe (DNA synthesis, Moscow, Russia), together with 5 μL cleaved sample DNA. The PCR designs were duplexed, combining each HPV genotype (16 and 18) with the human control *HBB* gene [12]. For the analysis of the studied genes, primers were selected for the main HPV oncogenes: E1/E2 and E6/E7 (Supplement Figure S1). The sequence of primers and probes was selected using the Vector NTI 11.5 program (Thermo Scientific, Waltham, MA, USA) using the genetic database www.ncbi.nlm.nih.gov (accessed on 1 November 2022), as well as the Primer-BLAST database (Supplement Table S1). The entire reaction mixture was loaded into a disposable plastic cartridge (Bio-Rad, Hercules, CA, USA) together with 70 μL of droplet generation oil (Bio-Rad, Hercules, CA, USA) and placed into the droplet generator (Bio-Rad, Hercules, CA, USA). After processing, the droplets generated from each sample were transferred to a 96-well PCR plate (Eppendorf, Hamburg, Germany). PCR amplification was carried out on a T100 Touch thermal cycler (Bio-Rad, Hercules, CA, USA) using a thermal profile beginning at 95 °C for 10 min, followed by 45 cycles of 94 °C for 10 s and 57 °C for 60 s, and ending at 98 °C for 10 min at a ramp rate of 2 °C/s. After PCR, the plate was loaded on the droplet reader (Bio-Rad, USA), and the acquired data were analyzed with QuantaSoft Analysis Pro software Version 1.7.4.0917 (Bio-Rad, Hercules, CA, USA).

### 2.5. Statistical Methods

Statistical data processing was carried out using the Statistica 8.0 (StatSoft Inc., Tulsa, OK, USA) software package. The incidence of HPV-positive and HPV-negative patients in each group was assessed in absolute values and as a percentage of the total number of patients studied. Fisher’s two-sided test was used to compare the incidence of HPV-positive patients in the groups depending on clinical and pathological parameters and response to NAC, as well as comparison with the control group of patients. Survival was assessed using the Kaplan–Meier method. The reliability of differences between the groups was compared using the log-rank test. The difference was considered reliable at *p* < 0.05.

## 3. Results

**Figure 1:**
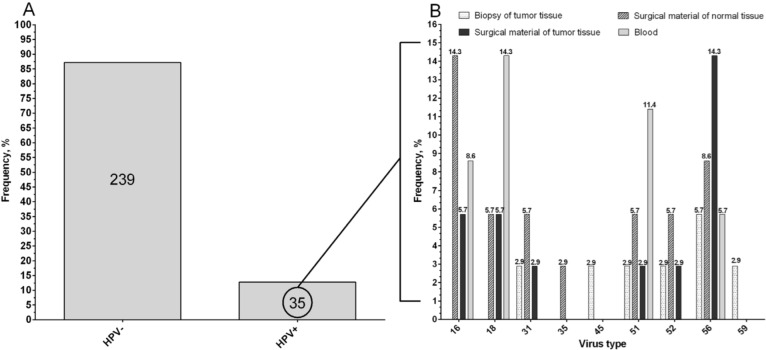
The frequency of types of HPV-positive patients (**A**), depending on the test material (**B**).

HPV was identified in 35 patients (12.7%) regardless of the group of patients and the type of material (Figure 1A). In the first group of patients with NAC, the presence of HPV was detected in only 9.5% of cases (12 of 126 patients). In the second group of patients under study, HPV was identified in 15.5% of patients (23 of 148 cases).

The HPV high-carcinogenic risk typing in the study group of patients detected nine types of the virus in patients with NSCLC: 16, 18, 31, 35, 45, 51, 52, 56, and 59. The frequencies of the types in HPV-positive patients, depending on the test material, are shown in Figure 1B. Most often, HPV was detected in the operative material of normal and tumor lung tissue as well as in the blood of the patients under study.

Mono- and mixed infections (the presence of two or more types of HPV at the same time) were observed in 23 and 12 patients, respectively (65.7% and 34.7%) (Table 2).

The analysis of the viral load of HPV-positive patients showed a predominantly insignificant viral load: 25 cases out of 35 were positive (74.3%). Six HPV-positive patients (17.1%) had a significant viral load, and there were four patients with an increased viral load (11.4%). The division into groups also showed that an insignificant viral load prevailed in the group of patients with NAC (66.7%) and the group of patients with primary operable LC (78.3%) (Table 2). Next, physical status was assessed for virus types 16, 18, and 45 (Table 2). Of all HPV-positive specimens, virus types 16, 18, and 45 were identified in 18 patients. Ten out of eighteen patients had an integrated form of the virus (55.6%), five out of eighteen patients (27.8%) had a free or episomal form, and three patients had a mixed form. It is important to note that the integrated form of the virus was identified in tissue samples, whereas the episomal and mixed forms were identified in the blood of the examined patients.

At the next stage, the association of clinical and pathological parameters (age, gender, tumor size, lymphogenous metastasis, stage, clinical and anatomical form, and histological type of tumor) with the presence of HPV and its virological parameters in patients with LC was assessed (Supplement Table S2). The incidence of HPV-positive patients in the overall patient group was found to be statistically significantly higher in the group with no lymphogenous metastasis (18.6%, 19/102 cases), compared with the HPV-negative patient group (9.3%, 16/172 cases) (*p* = 0.03). A similar result was shown in the group of patients with primary operable LC: 26% of HPV-positive patients versus 10.2% (*p* = 0.01). For the first group of patients, with NAC, there was a relationship between the presence of HPV and the tumor size. Interestingly, the presence of HPV was associated with a larger primary tumor node size (11 of 81 cases (13.6%) vs. 1 of 45 cases (2.2%) in the group with T_1–2_; *p* = 0.05). An association with a strong tendency was also demonstrated: HPV-positive patients were predominantly male (11.5%, 28 of 243 patients), which can be explained by the predominance of men in our sample of patients (88.7%). An analysis of the relationship between viral load and the main clinical and pathological parameters of patients showed that in 85.7% of cases (18 out of 21 patients) with a peripheral, clinical, and anatomical form of the tumor, an insignificant viral load prevailed (*p* = 0.05) (Supplement Table S3). In addition, patients in the group with tumor size T_3–4_, in 66.7% of cases, had an integrated physical form of the virus, whereas the episomal form was determined in patients with T_1–2_ in 100% of cases (*p* = 0.02) (Supplement Table S3).

**Figure 2:**
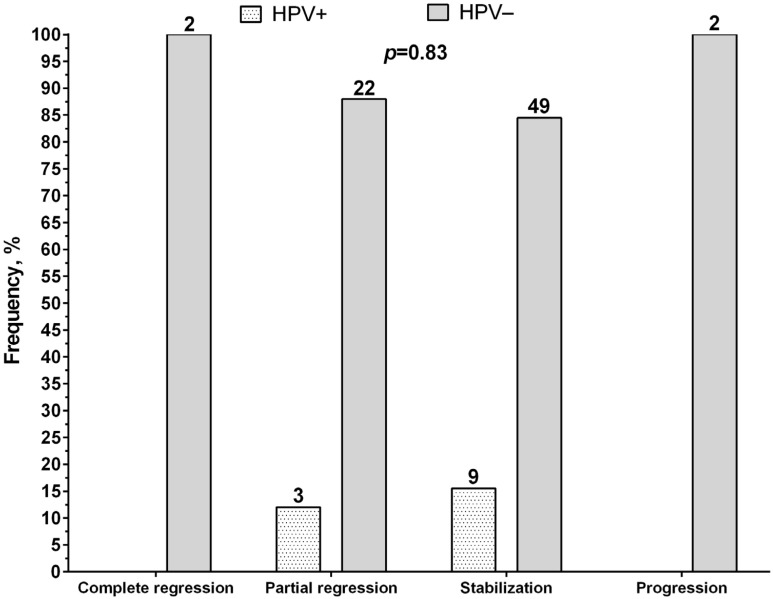
Association between the presence of HPV and the effectiveness of neoadjuvant chemotherapy in patients with lung cancer.

It is interesting to assess the association of HPV with the efficacy of neoadjuvant chemotherapy (Figure 2). Data on the effect of NAC were available for 87 patients with NSCLC, showing no HPV-positive patients with complete regression or progression. Human papillomavirus was detected in three patients with partial regression (12%) and in nine patients with tumor nodule stabilization (15.5%). A comparative analysis showed no association between HPV and the effectiveness of chemotherapy (*p* = 0.83).

**Figure 3:**
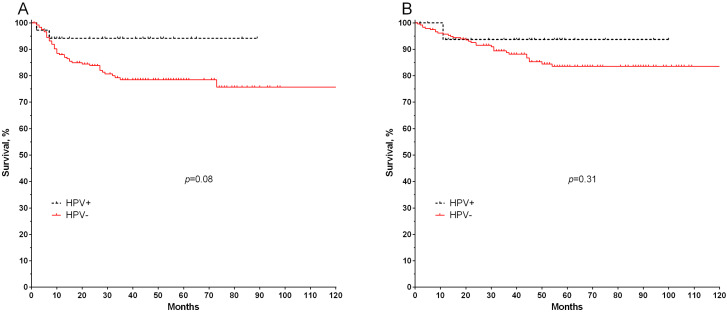
Metastasis-free (**A**) and overall survival (**B**) of HPV-positive and HPV-negative patients with lung cancer.

In the control group (*n =* 30), HPV type 18 was found in only one patient with a significant viral load and a mixed form. The association of HPV with lung cancer was then assessed using a two-tailed Fisher test (Figure 3A).

There was no association between HPV and LC (Table 3).

Thus, the incidence of HPV-positive specimens in the control group was 3.3% (1 of 30 cases), compared with the study group, where the rate was 12.7% (35 of 274 cases). The analysis performed showed no statistically significant differences in the presence of HPV in the study group compared with the control group (*p* = 0.49).

Many authors use normal tissue from the same patients as a control group when evaluating the association of HPV with lung cancer. Based on this, we performed an additional analysis of the association of HPV with lung cancer with the results of the analysis of normal lung tissue used as a control group (Table 3). It was shown that the frequency of HPV presence in normal lung tissue (4%, 11/274 patients) was the same as compared to tumor tissue (*p* = 1.00). In addition, no statistically significant relationship was shown for the blood of the studied patients (*p* = 0.37).

Then, the metastatic-free survival (MFS) of patients with lung cancer was assessed for HPV infection with a high carcinogenic risk. It was found that HPV-positive patients had a higher 5-year metastatic-free survival rate (94%) compared with HPV-negative patients (78%; Figure 3A). It can be assumed that the differences are not statistically significant due to the small number of HPV-positive patients, although the data obtained show a pronounced tendency (log-rank test, *p* = 0.08). The presence of HPV is not associated with overall survival (log-rank test, *p* = 0.31; Figure 3B).

In addition, a multivariate regression analysis was performed to identify prognostic factors for metastasis-free survival (Table 4).

It was found that the presence of HPV in patients with lung cancer is not a factor that increases or reduces the risk of tumor metastasis (HR = 0.91; 95% CI: 0.37–2.22; *p* = 0.83). A small tumor size (T_2_) in patients is a favorable factor for metastasis-free survival rates (HR = 0.16; 95% CI: 0.03–0.76; *p* = 0.02).

**Figure 4:**
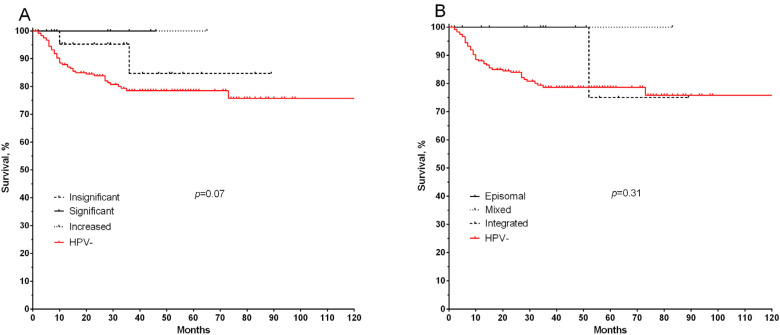
Metastasis-free survival of HPV-positive patients depends on the viral load (**A**) and the physical status of the virus (**B**).

Next, we also evaluated the association of virological parameters in HPV-positive patients with metastatic-free survival rates (Figure 4). It was found that patients with a significant and increased viral load had 100% survival rates, compared with patients with an insignificant load and HPV-negative patients. The result is not statistically significant, but a pronounced trend is shown at *p* = 0.07 (Figure 4A). At the same time, there were no statistically significant differences in the rates of non-metastatic survival according to the physical status of the studied virus (*p* = 0.31) (Figure 4B).

Thus, HPV-positive patients with NSCLC had a high level of metastatic survival in comparison with the survival rates of HPV-negative patients. It is important to note that the data obtaining a favorable outcome for patients with HPV-positive lung cancer, despite the small group size, not only agree with the global literature data but are also important for the prognosis of the disease.

## 4. Discussion

The etiological role of HPV in the development of anogenital carcinomas has been widely studied and is well known [13,14]. However, the role of this virus in the development of non-small cell lung cancer is controversial and is currently under discussion. There are few reports in the literature on the prevalence of HPV in NSCLC, and these are limited to a few countries in Europe, Asia, and parts of South America. To the best of our knowledge, this is the first study conducted in Russia; it aimed to assess the relationship between the characteristics of the spread of HPV infections in NSCLC and predict the outcome of patients. 

In this work, 12.7% of the cases of NSCLC were HPV-positive (35/274 patients). The data obtained were consistent with the results of our earlier meta-analysis, according to which the frequency of HPV in tumor tissues in LC was 28.7% and the frequency of HPV in normal lung tissues was 7.6% [8]. In total, nine types of HRC HPV were identified (16, 18, 31, 35, 45, 51, 52, 56, and 59), and a monoinfection was detected in 65.7% of HPV-positive patients.

Regarding the determination of the prevalence of the desired virus in normal and tumor tissues, we obtained figures of 31.4% and 22.9%, respectively. This result was consistent with the literature data, in particular in a 2020 study that included 140 patients with primary lung cancer, where it was shown that the total infection rate of the sample included in the study was 20.7%. The virus was detected in 9.3% of the tumor samples (13 out of 140) and in 16 paired samples of normal and tumor tissues (11.4%) [15]. 

It is important to note that for the samples of normal tissue, tumor tissue, and blood obtained from each individual patient, the highest percentage of virus identification was observed in the blood (40.0%). These data are unique thus far; the Russian literature does not mention the determination of HPV in the blood of patients with NSCLC. It is believed that HPV is spread through respiratory secretion through close contact with mucous membranes or the skin [16]. There is evidence that HPV can be carried by peripheral blood mononuclear cells [17]. The results of the determination of HPV HRC in the blood of patients with NSCLC were consistent with the literature data. In a study by Chiou et al., it was shown that the prevalence of HPV types 16/18 in the blood of patients with lung cancer was significantly higher than in the control group (47.7% vs. 12.6% for HPV 16, *p* < 0.0001; 30.9% vs. 5.2% in the control group for HPV 18, *p* < 0.0001) [18].

When determining the types of HRC HPV in the study sample, it was shown that the highest frequency of occurrence was characteristic of HPV types 16 and 18, with their predominance in the material of normal lung tissue and blood, respectively. A high frequency of HPV type 56 in the tumor tissue was also established. However, the leader in the frequency of occurrence was HPV genotype 18 (36.8%), which was consistent with the world literature data on the definition of HRC HPV among the oncopathologies of various localizations [19,20]. In a sample of 140 patients with primary lung cancer, it was shown that in 16 paired samples of normal and tumor tissues, 7 cases of HPV type 16 were detected, 4 with HPV type 18, 3 with HPV type 42, 1 with the HPV 6 genotype, and 1 sample with a mixed infection with HPV 18 and 33 genotypes [15]. In another study, it was shown that in the study of 102 samples of lung tumor tissues and 48 control samples, the presence of HPV DNA was determined in 52.9% (54/102) of cases and in 25% of cases (12/48), respectively. At the same time, the most common genotype in lung tumor tissue samples, with a frequency of 38.8%, was type 16 of the virus (21/54 cases) [11]. Patients with HPV type 16 have been shown to have an increased risk of developing lung cancer (95% CI 3.7–11.3; *p* < 0.0001) and HPV type 18 (95% CI 4.2–20.2; *p* < 0.0001) [11].

An important limitation of the presented work was the lack of information on the smoking status of the studied patients, since it has been shown that smoking is one of the main factors in the development of lung cancer. Although a recent study showed that HPV is found in 20% of non-smokers with lung cancer [21].

Regarding the analysis of HPV DNA concentrations in LC samples, one of the first studies of the significance of the viral load as a predictor of the development of this oncopathology was conducted by Coissard et al. The authors showed a low viral load of fewer than one copy of the virus per cell [22]. A few authors believe that even a low viral load (1–2 copies per cell) is sufficient to initiate a carcinogenic process in lung tissue [23]. In the present study, it was determined that 28.6% of HPV-positive patients had a clinically significant viral load using a standard approach to determining the concentration of HPV DNA. In de Oliveira et al., lung tumor samples were analyzed for the presence of the expression of E6 and E7 oncoproteins using immunohistochemical staining to assess viral activity. Both oncoproteins were observed in the nucleus and in the cytoplasm, indicating that HPV was active and probably induced the cell transformation. Thus, the presence of these oncoproteins indicates that HPV is not in a latent form in the lung tissue but actively expresses oncoproteins and probably causes cellular changes [24].

Several mechanisms have been proposed for HPV oncogenesis, including the presence of mutations and structural changes in genes. For example, several HPV-associated tumors contain an amplification of the proliferation and cell cycle regulator gene *E2F1* (20q1). Another important structural change in HPV infections is the deletion of the *TRAF3* gene, which occurs in approximately 20% of HPV-associated tumors. This gene plays an important role in antiviral immunity and is a regulator of *NF-κB* (cell growth and proliferation) [25]. In response to viral infections, the generation of a pro-inflammatory response includes the activation of numerous transcription factors, including *NF-κB*, and the secretion of numerous pro-inflammatory cytokines and metabolites, including *TGF-β*, *IL-1*, *IL-6*, *IL-11*, and *TNF-a*. This pro-inflammatory tissue microenvironment leads to the suppression of anti-humoral immunity and promotes tumor development and metastasis [26]. Presumably, HPV may play a role in *NF-κB* activation [27]. In non-small cell lung cancer cells, *NF-κB* activity is increased. Previous studies have shown that *NF-κB* acts as a tumor promoter in NSCLC. Elevated levels of *NF-κB* expression were found in NSCLC tissues in contrast to the corresponding normal tissues. *NF-κB* overexpression has been associated with metastasis and a poor prognosis for NSCLC patients [28]. Unfortunately, our study lacks additional molecular genetic data on patients, such as the mutation load of the KRAS, P53, EGFR, and PIK3CA genes, which may also be involved in the process of LC carcinogenesis. In the future, it seems interesting and necessary to study this area and correlate the obtained data with HPV.

Regarding the prediction of the outcome of patients with HPV-associated carcinomas, differences in the survival of patients with tumor pathologies of the reproductive organs [10,29,30], head and neck squamous cell carcinomas [31,32,33] and colorectal cancer [20] have been clearly shown. However, the relationship between HPV infections in LC and survival is still uncertain and controversial. We showed that, in the presence of HPV, the 5-year incidence of MFS was 94%. However, according to Cox’s multivariate regression analysis, HPV was not a factor increasing or decreasing the risk of tumor metastasis (OR = 0.91; 95% CI: 0.37–2.22; *p* = 0.83). The data available in the literature are consistent with the results obtained in this work. For example, Wang et al. showed that patients with HPV types 16 and 18 and lung adenocarcinomas had higher survival rates compared with patients without the virus in the tumor [34]. Ragin and others have also shown that the presence of the virus in tumor tissues is associated with a good prognosis for the disease [34]. It is important to note that not all authors confirmed the presence of HPV in patients with NSCLC; thus, its role in the occurrence and progression of this type of tumor was not considered [35,36].

To date, the role of HPV in the pathogenesis and outcome of patients with NSCLC remains debatable. 

This study investigates potential associations between HPV and NSLC in a cohort of patients with the presence of three study points for each patient, namely, normal tissue (control), tumor tissue, and blood. The only limiting factor was the small size of the total sample and the sample of HPV-positive patients. This study was designed and conducted to obtain data on the prevalence of HPV in patients with NSCLC. In the future, we aim to conduct a large-scale multicenter study to confirm the role of HRC HPV in the development and prognosis of NSCLC.

## Figures and Tables

**Table 1:** The main clinical and pathological parameters of patients.

Clinical and Pathological Parameter		Number of NAC Patients (abs., %), (*n* = 126)	Number of Patients with Primary Operable LC (abs., %), (*n* = 148)	Total Number of Patients (abs., %), (*n* = 274)
Age	≤50 years	15 (11.9)	20 (13.5)	35 (12.8)
	>50 years	111 (88.1)	128 (86.5)	239 (87.2)
Gender	Male	115 (91.3)	128 (86.5)	243 (88.7)
	Female	11 (8.7)	20 (13.5)	31 (11.3)
Tumor size	T_1_	9 (7.1)	23 (15.5)	32 (11.7)
	T_2_	36 (28.6)	60 (40.5)	96 (35.0)
	T_3_	59 (46.8)	55 (37.2)	114 (41.6)
	T_4_	22 (17.5)	10 (6.8)	32 (11.7)
Lymphogenous metastasis	N_0_	52 (41.3)	50 (51.4)	102 (37.2)
	N_1_	30 (23.8)	16 (10.8)	46 (16.8)
	N_2_	38 (30.2)	68 (45.9)	106 (38.7)
	N_3_	6 (4.8)	14 (9.5)	20 (7.3)
Stage	IIB	46 (36.5)	79 (53.4)	125 (45.6)
	IIIA	80 (63.5)	69 (46.6)	149 (54.4)
Clinical and anatomical form	Central	71 (56.3)	47 (31.8)	118 (43.1)
	Peripheral	55 (43.7)	101 (68.2)	156 (56.9)
Histological type	Squamous	91 (72.2)	77 (52.0)	168 (61.3)
	Adenocarcinoma	35 (27.8)	71 (48.0)	106 (38.7)
Type of operation	Pneumonectomy	46 (36.5)	47 (31.8)	93 (33.9)
	Lobectomy	80 (63.5)	101 (68.2)	181 (66.1)
Hematogenous metastasis	Yes	35 (27.8)	17 (11.5)	52 (19.0)
	No	91 (72.2)	131 (88.5)	222 (81.0)

Note: *n*—number of patients; T—tumor; N—nodus; NAC—neoadjuvant chemotherapy; LC—lung cancer.

**Table 2:** HPV positive samples, virus types, viral load, and physical status (for 16, 18, and 45 types) in patients with non-small cell lung cancer.

№	Patient	Biopsy of Tumor Tissue	Surgical Material from Normal Tissue	Surgical Material from Tumor Tissue	Blood	Viral Load	Physical Status
Group of patients with NAC							
1	A1	-	-	-	51	Insignificant	
2	Ch1	-	-	16, 56	-	Insignificant	Integrated
3	D1	45, 59	-	-	-	Significant	Integrated
4	D2	-	-	-	51	Insignificant	
5	G1	31, 56	-	-	-	Significant	
6	H1	-	-	-	56	Insignificant	
7	K1	-	16, 18	-	-	Insignificant	Integrated
8	K2	-	-	-	16	Increased	Episomal
9	K3	51, 52, 56	-	-	-	Insignificant	
10	P1	-	16	-	-	Insignificant	Integrated
11	R1	-	56	-	-	Significant	
12	U1	-	16	-	-	Insignificant	Integrated
Number of patients (*n* = 12)		3	4	1	4		
Group of patients with primary operable LC							
13	B1		31	-	-	Increased	
14	B2		-	18, 31	-	Insignificant	Integrated
15	B3		35, 52	52	-	Increased	
16	B4		-	-	18	Insignificant	Episomal
17	B5		-	56	-	Insignificant	
18	Ch2		51, 56	-	-	Insignificant	
19	D2		52	-	-	Significant	
20	H1		-	16	-	Insignificant	Integrated
21	I1		-	-	16	Insignificant	Mixed
22	K4		-	-	18	Insignificant	Episomal
23	K5		-	-	18	Insignificant	Episomal
24	K6		-	-	18	Insignificant	Mixed
25	K7		16	-	-	Insignificant	Integrated
26	M1		51, 56	-	-	Insignificant	
27	M2		-	-	51	Insignificant	
28	N1		-	-	18	Insignificant	Episomal
29	O1		-	51, 56	-	Insignificant	
30	P2		-	-	56	Insignificant	
31	R2		16, 18, 31, 51	-	-	Significant/Insignificant (51 type)	Integrated (16 type)/Mixed (18 type)
32	R3		-	18, 56	-	Insignificant	Integrated
33	T1		-	-	51	Insignificant	
34	V1		-	56	-	Significant	
35	V2		-	-	16	Increased	Mixed
Number of patients (*n* = 23)		0	7	7	10		

Note: NAC—neoadjuvant chemotherapy; LC—lung cancer.

**Table 3:** Comparative analysis of the presence of HPV in the study groups and the control group.

Groups	Total Number of Patients (abs., %), (*n =* 274)		Number of NAC Patients (abs., %), (*n =* 126)		Number of Patients with Primary Operable LC (abs., %), (*n =* 148)	
	Study group—presence of HPV in tumor and normal lung tissue/Control group—bronchoalveolar lavage					
	HPV+	HPV−	HPV+	HPV−	HPV+	HPV−
Study group	21 (7.7)	253 (92.3)	8 (6.3)	118 (93.7)	14 (3.5)	134 (90.5)
Control group	1 (3.3)	29 (96.7)	1 (3.3)	29 (96.7)	1 (3.3)	29 (96.7)
*p*-level	0.49		0.69		0.32	
	Study group—presence of HPV in tumor tissue of the lung/Control group—normal lung tissue					
	HPV+	HPV−	HPV+	HPV−	HPV+	HPV−
Study group	11 (4.0)	263 (96.0)	4 (3.2)	122 (96.8)	7 (4.7)	141 (95.3)
Control group	11 (4.0)	263 (96.0)	4 (3.2)	122 (96.8)	7 (4.7)	141 (95.3)
*p*-level	1.00		1.00		1.00	
	Study group—presence of HPV in tumor tissue of the lung/Control group—normal lung tissue					
	HPV+	HPV−	HPV+	HPV−	HPV+	HPV−
Study group	14 (1.1)	260 (94.9)	4 (3.2)	122 (96.8)	10 (6.8)	138 (93.2)
Control group	0 (0.0)	30 (100.0)	0 (0.0)	30 (100.0)	0 (0.0)	30 (100.0)
*p*-level	0.37		0.58		0.21	

Note: NAC—neoadjuvant chemotherapy; LC—lung cancer. Statistically significant differences are in bold.

**Table 4:** Multivariate Cox regression analysis for metastasis-free survival (MFS) of patients in the study group.

Factor	MFS	
	HR (95% CI)	*p*-Value
Gender		
Male	1.00	
Female	0.92 (0.38–2.22)	0.86
Age		
>50	1.00	
≤50	0.79 (0.33–1.90)	0.60
Tumor size		
T_1_	1.00	
T_2_	0.16 (0.03–0.76)	0.02
T_3_	0.50 (0.21–1.20)	0.12
T_4_	0.62 (0.28–1.36)	0.23
Lymphogenous metastasis		
N_0_	1.00	
N_1_	0.38 (0.08–1.80)	0.22
N_2_	0.39 (0.07–2.07)	0.27
N_3_	0.97 (0.22–4.33)	0.97
Clinical and anatomical form		
Central	1.00	
Peripheral	1.19 (0.64–2.25)	0.57
Histological type of the tumor		
Squamous cell carcinoma	1.00	
Adenocarcinoma	1.18 (0.63–2.23)	0.60
Nature of surgery		
Lobectomy	1.00	
Pneumonectomy	1.22 (0.62–2.37)	0.55
HPV status		
HPV positive	1.00	
HPV negative	0.91 (0.37–2.22)	0.83

Note: T—tumor; N—nodus; HPV—human papillomavirus; MFS—metastasis-free survival.

## Data Availability

1. Database No. 2022621329 dated 6 June 2022 “Database of human papillomavirus infection in patients with non-small cell lung cancer with various types of precancerous changes in the bronchial epithelium” Tsyganov M.M., Ibragimova M.K., Khozyainova A.A., Gerashchenko T.S., Pankova O.V., Tashireva L.A., Zdereva E.A., Rodionov E.O., Miller V.S., Tuzikov S.A., Cheremisina O.V., Perelmuter V.M., Litvyakov N.V. 2. Database No. 2022621319 dated 3 June 2022 “Database of human papillomavirus infection in patients with stage IV-III non-small cell lung cancer” Tsyganov M.M., Ibragimova M.K., Rodionov E.O., Miller V.S., Tsydenova I.A., Gaptulbarova K.A., Dolgasheva D.S., Zdereva E.A., Tuzikov S.A., Cheremisina O.V., Litvyakov N.V. 3. Database No. 2022621373 dated 10 June 2022 “Database of the outcome of patients diagnosed with non-small cell lung cancer, taking into account infection with the human papillomavirus and its virological parameters” Tsyganov M.M., Ibragimova M.K., Rodionov E.O., Miller V.S., Tsydenova I.A., Gaptulbarova K.A., Dolgasheva D.S., Zdereva E.A., Tuzikov S.A., Cheremisina O.V., Litvyakov N.V.

## Supplementary Materials

The following supporting information can be downloaded at: https://www.mdpi.com/article/10.3390/microorganisms11010212/s1, Table S1: Sequence of the primers and probes used in the study; Figure S1: The result of ddPCR to assess the presence of HPV in the studied samples: viral copies per microliter for HPV 16 (A) and HPV 18 (B); Table S2: Relationship between the presence of HPV and the main clinical and pathological parameters of patients with non-small cell lung cancer; Table S3: Relationship of HPV viral load in patients with the main clinical and pathological parameters; Table S4: Relationship of the physical status of HPV (for types 16, 18 and 45) with the main clinical and pathological parameters.
