# Supplementary material for: Human Papillomavirus in Non-Small Cell Lung Carcinoma: Assessing Virus Presence in Tumor and Normal Tissues and Its Clinical Relevance
Source: Microorganisms. 2023 Jan 14;11(1):212. doi: 10.3390/microorganisms11010212 (PMC9865181; doi:10.3390/microorganisms11010212)
Supplement: Supplementary file 1 [file microorganisms-11-00212-s001.zip › Supplement 1.pdf]

Table S1. Sequence of the primers and probes used in the study

Genes		Amplicon (bp)	Sequence
HBB		124 bp	F 5'-gccagccgagccacatc-3'
			R 5'-ggcaacaatatccactttaccaga-3'
			Probe FAM 5'-cgcccaatacgaccaaatccg-3' BHQ1
HPV16	*E1*	73 bp	F 5'-gagaagatgacccagatcatgtt-3'
			R 5'-atagcacagcctggatagcaa-3'
			Probe HEX 5'-agaccttcaacaccccagccat-3' BHQ2
	E2	94 bp	F 5'-actaagcaccctgactatgctatcc-3'
			R 5'-cttccatcacatcactgaacacttt-3'
			Probe HEX 5'-cagccaggatcgctgtctctaacttgca-3' BHQ2
	E6	121 bp	F 5'-ggcgacgtaattcccgacta-3'
			R 5'-agttcttccccaggctctgc-3'
			Probe HEX 5'-accacaacctgcacccagactacatcca-3' BHQ2
	E7	107 bp	F 5'-acagctgtgtggtgcttctgtg-3'
			R 5'-cattgtcctctgtccaggcatc-3'
			Probe HEX 5'-catcattcacccttggcacaggtgt-3' BHQ2
HPV18	*E1*	73 bp	F 5'-gagaagatgacccagatcatgtt-3'
			R 5'-atagcacagcctggatagcaa-3'
			Probe HEX 5'-agaccttcaacaccccagccat-3' BHQ2
	E2	94 bp	F 5'-actaagcaccctgactatgctatcc-3'
			R 5'-cttccatcacatcactgaacacttt-3'
			Probe HEX 5'-cagccaggatcgctgtctctaacttgca-3' BHQ2
	E6	121 bp	F 5'-ggcgacgtaattcccgacta-3'
			R 5'-agttcttccccaggctctgc-3'
			Probe HEX 5'-accacaacctgcacccagactacatcca-3' BHQ2
	E7	107 bp	F 5'-acagctgtgtggtgcttctgtg-3'
			R 5'-cattgtcctctgtccaggcatc-3'
			Probe HEX 5'-catcattcacccttggcacaggtgt-3' BHQ2

Note: bp - base pair; F - forward primer; R - reverse primer.

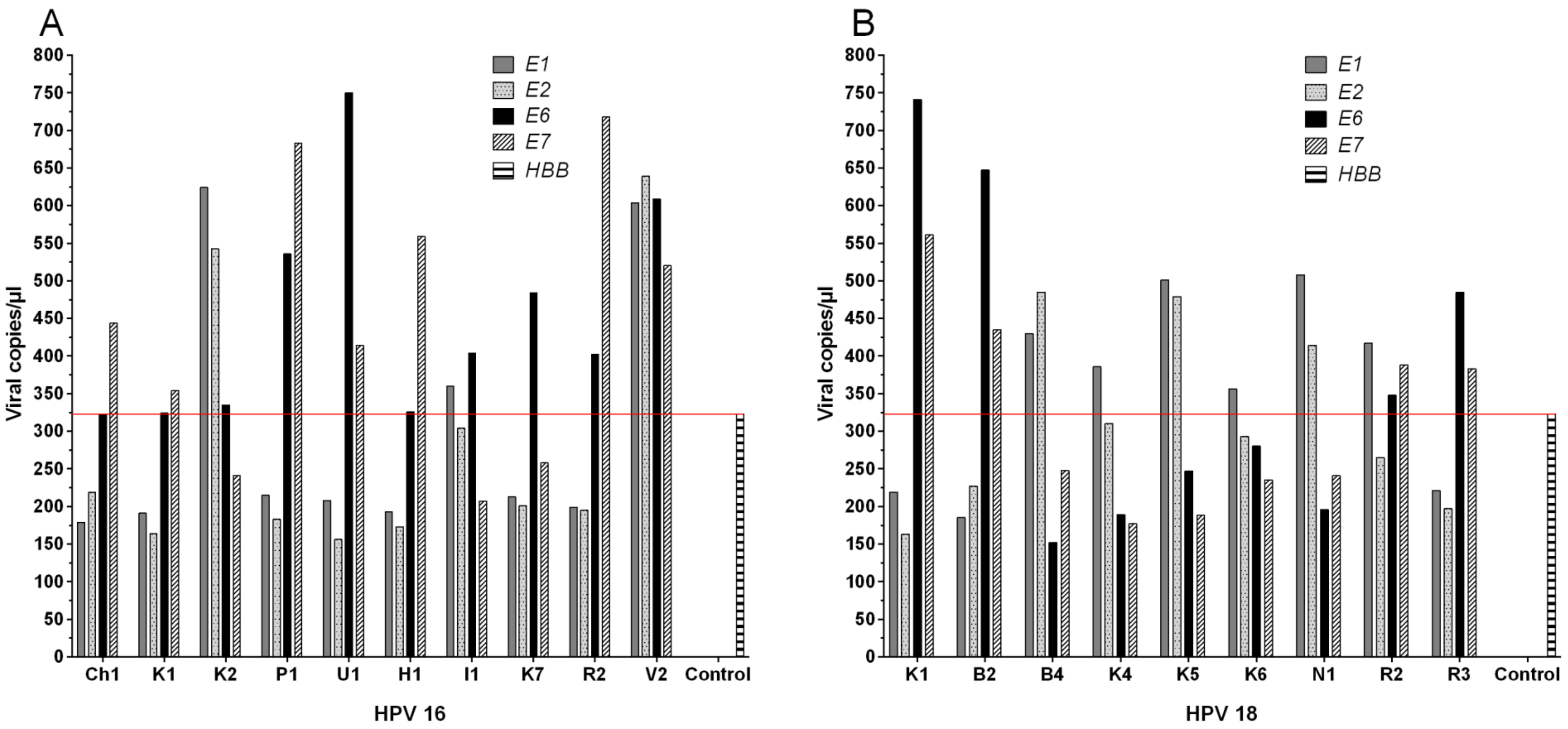

Figure S1. The result of ddPCR to assess the presence of HPV in the studied samples: viral copies per microliter for HPV 16 (A) and HPV 18 (B).
