# Supplementary material for: Human Papillomavirus in Non-Small Cell Lung Carcinoma: Assessing Virus Presence in Tumor and Normal Tissues and Its Clinical Relevance
Source: Microorganisms. 2023 Jan 14;11(1):212. doi: 10.3390/microorganisms11010212 (PMC9865181; doi:10.3390/microorganisms11010212)
Supplement: Supplementary file 1 [file microorganisms-11-00212-s001.zip › Supplement 2.pdf]

Supplement 2

Table S2. Relationship between the presence of HPV and the main clinical and pathological parameters of patients with non-small cell lung cancer.

Clinical and pathological parameter		Total number of patients (abs., %). (n=274)			Number of NAC patients (abs., %). (n=126)			Number of Patients with primary operable LC (abs., %). (n=148)		
		HPV+	HPV-	p-level	HPV+	HPV-	p-level	HPV+	HPV-	p-level
Age	≤50 years	4 (11.4)	31 (88.6)	1	2 (13.3)	13 (86.7)	0.63	2 (10.0)	18 (90.0)	0.53
	>50 years	31 (13.0)	208 (87.0)		10 (9.0)	101 (91.0)		21 (16.4)	107 (83.6)	
Gender	Male	***28 (11.5)***	***215 (88.5)***	***0.09***	10 (8.7)	105 (91.3)	0.59	18 (14.1)	110 (85.9)	0.31
	Female	***7 (22.6)***	***24 (77.4)***		2 (18.2)	9 (81.8)		5 (25.0)	15 (75.0)	
Tumor size	T_{1-2}	12 (9.4)	116 (90.6)	0.14	**1 (2.2)**	**44 (97.8)**	**0.05**	11 (13.3)	72 (86.7)	0.49
	T_{3-4}	23 (15.8)	123 (84.2)		**11 (13.6)**	**70 (86.4)**		12 (18.5)	53 (81.5)	
Lymphogenous metastasis	N_0	**19 (18.6)**	**83 (81.4)**	**0.03**	6 (11.5)	46 (88.5)	0.55	**13 (26.0)**	**37 (74.0)**	**0.01**
	N_{1-3}	**16 (9.3)**	**156 (90.7)**		6 (8.1)	68 (91.9)		**10 (10.2)**	**88 (89.8)**	
Stage	IIB	14 (11.2)	111 (88.8)	0.58	3 (6.5)	43 (93.5)	0.53	11 (13.9)	68 (86.1)	0.65
	IIIA	21 (14.1)	128 (85.9)		9 (11.3)	71 (88.8)		12 (17.4)	57 (82.6)	
Clinical and anatomical form	Central	14 (11.9)	104 (88.1)	0.71	9 (12.7)	62 (87.3)	0.22	5 (10.6)	42 (89.4)	0.33
	Peripheral	21 (13.5)	135 (86.5)		3 (5.5)	52 (94.5)		18 (17.8)	83 (82.2)	
Histological type	Squamous	18 (10.7)	150 (89.3)	0.26	8 (8.8)	83 (91.2)	0.73	10 (13.0)	67 (87.0)	0.49
	Adenocarcinoma	17 (16.0)	89 (84.0)		4 (11.4)	31 (88.6)		13 (18.3)	58 (81.7)	

Note: Bold indicates statistically significant differences; bold and italic - relationship at the level of a pronounced trend (p<0.1).

Table S3. Relationship of HPV viral load in patients with the main clinical and pathological parameters.

Clinical and pathological parameter		Total number of patients (abs., %). (n=35)				Number of NAC patients (abs., %). (n=12)				Number of Patients with primary operable LC (abs., %). (n=23)			
		Insignificant	Significant	Increased	p-level	Insignificant	Significant	Increased	p-level	Insignificant	Significant	Increased	p-level
Age	≤50 years	4 (100.0)	0 (0.0)	0 (0.0)	0.45	2 (100.0)	0 (0.0)	0 (0.0)	0.54	2 (100.0)	0 (0.0)	0 (0.0)	0.73
	>50 years	22 (71.0)	5 (16.1)	4 (12.9)		6 (60.0)	3 (30.0)	1 (10.0)		16 (76.2)	2 (9.5)	3 (14.3)	
Gender	Male	18 (66.7)	5 (18.5)	4 (14.8)	0.45	6 (60.0)	3 (30.0)	1 (10.0)	0.54	12 (70.6)	2 (11.8)	3 (17.6)	0.72
	Female	6 (85.7)	0 (0.0)	1 (14.3)		2 (100.0)	0 (0.0)	0 (0.0)		4 (80.0)	0 (0.0)	1 (20.0)	
Tumor size	T_{1-2}	9 (75.0)	1 (8.3)	2 (16.7)	0.75	1 (100.0)	0 (0.0)	0 (0.0)	0.75	8 (72.7)	1 (9.1)	2 (18.2)	0.99
	T_{3-4}	16 (69.6)	4 (17.4)	3 (13.0)		7 (63.6)	3 (27.3)	1 (9.1)		9 (75.0)	1 (8.3)	2 (16.7)	
Lymphogenous metastasis	N_0	13 (68.4)	4 (21.1)	2 (10.5)	0.78	***3 (50.0)***	***3 (50.0)***	***0 (0.0)***	***0.10***	10 (76.9)	1 (7.7)	2 (15.4)	0.93
	N_{1-3}	12 (75.0)	1 (6.3)	3 (18.8)		***5 (83.3)***	***0 (0.0)***	***1 (16.7)***		7 (70.0)	1 (10.0)	2 (20.0)	
Stage	IIB	10 (71.4)	2 (14.3)	2 (14.3)	1	2 (66.7)	1 (33.3)	0 (0.0)	0.80	8 (72.7)	1 (9.1)	2 (18.2)	0.99
	IIIA	15 (71.4)	3 (14.3)	3 (4.0)		6 (66.7)	2 (22.2)	1 (11.1)		9 (75.0)	1 (8.3)	2 (16.7)	
Clinical and anatomical form	Central	**7 (50.0)**	**4 (28.6)**	**3 (14.3)**	**0.05**	5 (55.6)	3 (33.3)	1 (11.1)	0.36	2 (40.0)	1 (20.0)	2 (40.0)	0.14
	Peripheral	**18 (85.7)**	**1 (4.8)**	**2 (21.4)**		3 (100.0)	0 (0.0)	0 (0.0)		15 (83.3)	1 (5.6)	2 (11.1)	
Histological type	Squamous	11 (61.1)	3 (16.7)	4 (22.2)	0.31	5 (62.5)	2 (25.0)	1 (12.5)	0.75	6 (60.0)	1 (10.0)	3 (30.0)	0.34
	Adenocarcinoma	14 (82.4)	2 (11.8)	1 (5.9)		3 (75.0)	1 (25.0)	0 (0.0)		11 (84.6)	1 (7.7)	1 (7.7)	

Note: Bold indicates statistically significant differences; bold and italic - relationship at the level of a pronounced trend (p<0.1).

Table S4. Relationship of the physical status of HPV (for types 16. 18 and 45) with the main clinical and pathological parameters.

Clinical and pathological parameter		Total number of patients (abs., %). (n=18)				Number of NAC patients (abs., %). (n=6)				Number of Patients with primary operable LC (abs., %). (n=12)			
		Episomal	Mixed	Integrated	p-level	Episomal	Mixed	Integrated	p-level	Episomal	Mixed	Integrated	p-level
Age	≤50 years	0 (0.0)	0 (0.0)	1 (100.0)	0.58	0 (0.0)	0 (0.0)	1 (100.0)	0.88	0 (0.0)	0 (0.0)	0 (0.0)	1
	>50 years	6 (35.3)	2 (11.8)	9 (52.9)		1 (20.0)	0 (0.0)	4 (80.0)		5 (41.7)	3 (25.0)	4 (33.3)	
Gender	Male	4 (30.8)	1 (7.7)	8 (61.5)	0.64	1 (20.0)	0 (0.0)	4 (80.0)	0.88	3 (37.5)	1 (12.5)	4 (50.0)	0.68
	Female	2 (40.0)	1 (20.0)	2 (40.0)		0 (0.0)	0 (0.0)	1 (100.0)		2 (50.0)	1 (25.0)	1 (25.0)	
Tumor size	T_{1-2}	**3 (100.0)**	**0 (0.0)**	**0 (0.0)**	**0.02**	0 (0.0)	0 (0.0)	0 (0.0)	1	***3 (100.0)***	***0 (0.0)***	***0 (0.0)***	***0.06***
	T_{3-4}	**3 (20.0)**	**2 (13.3)**	**10 (66.7)**		1 (16.7)	0 (0.0)	5 (83.3)		***2 (22.2)***	***2 (22.2)***	***5 (55.6)***	
Lymphogenous metastasis	N_0	4 (36.4)	1 (9.1)	6 (54.5)	0.64	0 (0.0)	0 (0.0)	3 (100.0)	0.54	4 (50.0)	1 (12.5)	3 (37.5)	0.68
	N_{1-3}	2 (28.6)	1 (14.3)	4 (57.1)		1 (33.3)	0 (0.0)	2 (66.7)		1 (25.0)	1 (25.0)	2 (50.0)	
Stage	IIB	3 (50.0)	1 (16.7)	2 (33.3)	0.40	0 (0.0)	0 (0.0)	1 (100.0)	0.88	3 (60.0)	1 (20.0)	1 (20.0)	0.42
	IIIA	3 (25.0)	1 (8.3)	8 (66.7)		1 (20.0)	0 (0.0)	4 (80.0)		2 (28.6)	1 (14.3)	4 (57.1)	
Clinical and anatomical form	Central	2 (28.6)	1 (14.3)	4 (57.1)	0.90	1 (20.0)	0 (0.0)	4 (80.0)	0.88	1 (50.0)	1 (50.0)	0 (0.0)	0.26
	Peripheral	4 (36.4)	1 (9.1)	6 (54.5)		0 (0.0)	0 (0.0)	1 (100.0)		4 (40.0)	1 (10.0)	5 (50.0)	
Histological type	Squamous	3 (30.0)	1 (10.0)	6 (60.0)	0.91	1 (25.0)	0 (0.0)	3 (75.0)	0.74	2 (33.3)	1 (16.7)	3 (50.0)	0.81
	Adenocarcinoma	3 (37.5)	1 (12.5)	4 (50.0)		0 (0.0)	0 (0.0)	2 (100.0)		3 (50.0)	1 (16.7)	2 (33.3)	

Note: Bold indicates statistically significant differences; bold and italic - relationship at the level of a pronounced trend ($p<0.1$).
